# A Noise-Robust Heart Sound Segmentation Algorithm Based on Shannon Energy

**DOI:** 10.1109/access.2024.3351570

**Published:** 2024-01-08

**Authors:** YOUNESS ARJOUNE, TRONG N. NGUYEN, ROBIN W. DOROSHOW, RAJ SHEKHAR

**Affiliations:** 1 Sheikh Zayed Institute for Pediatric Surgical Innovation, Children’s National Hospital, Washington, DC 20010, USA; 2 AusculTech Dx, Silver Spring, MD 20902, USA; 3 Department of Cardiology, Children’s National Hospital, Washington, DC 20010, USA

**Keywords:** Auscultation, heart sound segmentation, Shannon energy, CirCor DigiScope phonocardiogram dataset, artificial intelligence, deep learning, stethoscope, heart murmur classification

## Abstract

Heart sound segmentation has been shown to improve the performance of artificial intelligence (AI)-based auscultation decision support systems increasingly viewed as a solution to compensate for eroding auscultatory skills and the associated subjectivity. Various segmentation approaches with demonstrated performance can be utilized for this task, but their robustness can suffer in the presence of noise. A noise-robust heart sound segmentation algorithm was developed and its accuracy was tested using two datasets: the CirCor DigiScope Phonocardiogram dataset and an in-house dataset – a heart murmur library collected at the Children’s National Hospital (CNH). On the CirCor dataset, our segmentation algorithm marked the boundaries of the primary heart sounds S1 and S2 with an accuracy of 0.28 ms and 0.29 ms, respectively, and correctly identified the actual positive segments with a sensitivity of 97.44%. The algorithm also executed four times faster than a logistic regression hidden semi-Markov model. On the CNH dataset, the algorithm succeeded in 87.4% cases, achieving a 6% increase in segmentation success rate demonstrated by our original Shannon energy-based algorithm. Accurate heart sound segmentation is critical to supporting and accelerating AI research in cardiovascular diseases. The proposed algorithm increases the robustness of heart sound segmentation to noise and viability for clinical use.

## I. INTRODUCTION

Cardiovascular diseases remain the leading cause of death worldwide. The World Health Organization (WHO) reported 17.9 million deaths from cardiovascular diseases in 2016 and this number is expected to reach 23.6 million by 2030 [1], [2], [3], [4], [5]. These diseases also carry a high financial burden, which is projected to rise to $749 billion by 2035 [1]. Well-honed cardiac auscultation remains a low-cost screening tool for early detection of heart disease [6]. It is, however, subjective and unappreciated by many practitioners [7], [8]. Davidsen et al. [9] reported a high variability in the sensitivity and the accuracy of auscultation. Such variability leads to an increase in unnecessary referrals to expert cardiologists, overuse of echocardiography and other diagnostics, and potentially contributes to missed abnormalities [8]. In recent years, there has been a growing interest in empowering primary care providers, the so-called “gate-keepers” of the healthcare delivery system, with advanced auscultation tools. One way to address auscultation’s subjectivity and providers’ eroding skills is to build decision support systems, such as artificial intelligence (AI)-based auscultation [10], [11], [12]. Heart sound segmentation (HSS)–identification of the primary heart sounds (S1 and S2)–is often a prerequisite in computerized auscultation. It has been reported to be used in many AI-based heart murmur classification pipelines [10], [11], [12]. However, some deep-learning approaches skip this step. This raises an interesting research question: what is the incremental value of segmentation in enabling AI-based automated heart murmur identification, given that convolutional neural networks (CNNs) are powerful feature extraction tools? Oliveira et al. [13] showed that segmentation boosts the performance of deep-learning algorithms for heart murmur classification. In addition, HSS also provides a means to increase the size of the training datasets, which is helpful for data-intensive deep-learning algorithms. With segmentation as a first step, classification can be performed on individual cardiac cycles, and the majority vote or mean absolute value can be used for a recording-level classification [13], [14]. In the 348 open-source entries submitted to the 2016 PhysioNet Challenge by 48 teams, Clifford et al. [14] demonstrated that the methods that included segmentation achieved significant improvements in heart abnormality classification tasks. Seventeen of the 20 highest-scoring papers in the 2016 PhysioNet Challenge used segmentation (See Table 1). More recent papers also support this finding [10], [11], [15], [16], [17], [18], [19]. One could argue that segmentation is not the only reason why these methods performed better, but, given that the accuracies of the top six entries ranged within 2% despite using different classifiers, segmentation appears to be a dominant factor. In the context of the 2022 PhysioNet Challenge, HSS was not the primary focus of the challenge, but it was a critical preprocessing step for many of the algorithms submitted by the participating teams (Table 2).

Manual segmentation is an alternative but it is not practical in most situations given the size of the datasets required for deep learning. Moreover, intra- and inter-observer HSS variability would affect accuracy. Therefore, it is imperative to develop automated segmentation techniques. Many segmentation techniques have been proposed using different approaches. A review of segmentation techniques based on wavelet transform, fractal decomposition, Hilbert transform, and Shannon energy envelogram was published by Milani et al. [20]. The review showed that Shannon energy envelogram-based techniques generally achieved higher performance. Our team previously reported a heart sound segmentation algorithm using Shannon energy envelogram [15]. This algorithm was developed and tested using heart sound recordings collected with a commercial electronic stethoscope (Littmann Model 4100, 3M Company, St Paul, Minnesota, USA) and curated by a clinical expert to remove any noisy segments at the beginning or the end of the recording. The heart sound recordings can be corrupted by noise, including endogenous or ambient speech, motion artifacts, and physiological sounds such as intestinal and breath sounds, any of which could lead to incorrect segmentation or failure. A noise-robust algorithm that could segment a recording successfully even in the presence of noise is therefore highly desirable. This paper presents a noise-robust version of original HSS algorithm (referred to as the Shannon energy-based algorithm henceforth) and its validation using both in-house and independent third-party datasets. The third-party dataset, hereinafter termed the CirCor dataset, is a part of the 2022 PhysioNet challenge [21]. The remainder of this paper is organized as follows. Section II describes the segmentation algorithms, first the Shannon energy-based and then its proposed noise-robust version. Section III presents the results of applying segmentation to the two datasets. Section IV discusses the results in the context of related work demonstrating that the noise-robust segmentation algorithm led to better classification performance.

## II. RELATED WORK

The state-of-the-art heart sound segmentation algorithms can be grouped into seven classes: 1) wavelet transform [56]; 2) fractal decomposition [57]; 3) Hilbert transform [58]; 4) hidden semi-Markov model (HSMM); 5) deep learning [59]; 6) hybrid methods [60]; 7) and Shannon energy envelogram [61], [62]. The advantages and limitations of these techniques are summarized in Table 3. The table indicates that wavelet transform-based segmentation techniques split the phonocardiograms into frequency bands. They provide adequate performance, but have questionable performance with non-stationary signals. Fractal decomposition techniques do not need to split the phonocardiograms into frequency bands, as they work on time-domain signals. Fractal decomposition techniques can segment a heart sound recording with murmurs. Hilbert transform-based HSS techniques, like those based on wavelet transform, split the heart sound signal into frequency bands and work with individual bands. These techniques have an advantage over the latter as they indicate adequate performance on both stationary and non-stationary signals as they can detect even minimal changes in frequencies. The performance of these techniques on wideband signals, however, is insufficient. HSMM-based segmentation techniques are probabilistic statistical, stochastic state machines with hidden Markov processes. They are robust to variability between testing and training datasets, but these approaches are computationally intensive. Deep learning techniques provide adequate performance and are able to generalize on unseen datasets, but they require hard-to-develop large training datasets. Several stethoscopes can be used for data acquisition. Hardware bias from the variability in the frequency response of stethoscopes poses a challenge. Hybrid methods employ electrocardiography equipment and use Q, R, and S waves for HSS. The hardware and software requirements make hybrid methods less practical. These electrocardiography-based segmentation methods necessitate simultaneous recording and synchronous processing of both the heart sound and ECG signal, which can be inconvenient, particularly when dealing with infants or newborn children [63]. Despite these efforts, HSS remains especially challenging in pediatrics because of the higher heart rate in children. Some of the reported techniques assumed that the diastole is longer than the systole, which does not always hold in children. Shannon energy-based segmentation methods provide superior segmentation accuracy compared with prior techniques, are computationally efficient, diminish unwanted signal peaks, and work in time domain [20]. They do not require splitting the signal into frequency bands and can be used in conjunction with other methods for adaptive thresholding. Shannon energy-based HSS requires some threshold tuning and could be challenging because of the sensitivity to noise. The noise-robust segmentation algorithm presented here builds upon our original Shannon energy algorithm and introduces smart cropping to increase the robustness of these methods to noise.

## III. SEGMENTATION FRAMEWORK

This section describes the Shannon energy-based segmentation algorithm followed by the proposed noise-robust heart sound segmentation technique. The general block diagram is shown in Fig. 2.

### A. SHANNON ENERGY-BASED SEGMENTATION ALGORITHM

This algorithm uses Shannon energy to segment the primary heart sounds S1 and S2. After preprocessing heart sound recordings, the algorithm finds and validates the sound lobes by applying a priori knowledge.These steps are described here in brief; a detailed description of this algorithm is available in [15].

#### 1) PRE-PROCESSING

The first step in the phonocardiogram preprocessing is to resample the signal to 4,000 Hz. The next step filters out low and high frequency components not associated with heart sounds by applying a bandpass filter with 40 Hz and 500 Hz as lower and upper cutoff frequencies. The last step normalizes the band-pass filtered signal to [−1,1] range.

#### 2) IDENTIFICATION OF CANDIDATE SOUND LOBES

The next step identifies candidate sound lobes, for which the envelope signal is computed. The envelope signal *E*_*s*_ of the normalized band pass-filtered heart sound signal is computed as the average Shannon energy (ASE), taken at fixed intervals. ASE has been widely utilized for extracting the heart sound envelope in prior studies [67], [68], [69], [70]. The ASE is computed over 20-ms sliding windows with 50% overlap between two successive windows. Let $x_{\mathrm{norm}}(t)$ denote the normalized bandpass filtered heart sound signal. The ASE is computed as,

(1) $$E_{s}=\frac{-1}{N}\sum_{j=1}^{N}x_{norm}\left(j\right)\text{log}\;x_{norm}{\left(j\right)}^{2},$$

where N denotes the total number of samples in a 20-ms window. Then, the ASE is normalized over all time instants. The normalized ASE (NASE) is given as,

(2) $$NASE(t)=E_{S}(t)-\overset{-}{E_{S}}(t),$$

where $\overset{-}{E_{s}}(t)$ denotes the mean of $E_{s}(t)$. The sound lobe boundaries are localized by applying the threshold value of 0 on the NASE.

### B. HEART SOUND LOBE VALIDATION

The localized sound lobes are validated with the goal of removing extra sounds not corresponding to the primary heart sounds (S1 and S2). To achieve this, two conditions are applied, informed by a priori knowledge, on the sound lobe duration and the interval between adjacent lobes. First, both S1 and S2 are less than 250 ms in duration [70]. Moreover, each of these can be split into two sounds, and when that happens, the maximum split interval is generally no greater than 50 ms [69], [70] and the split sound lobes have lower intensity. To check if a split has occurred, the time interval between two such sound lobes is tested if it is less than 50 ms, and the root mean square (RMS) energy of one lobe is less than 40% of the other. If the split is present, the higher-energy sound lobe is kept. If they have similar energies, one of the two lobes could be a murmur or noise, in which case both sounds are retained as candidates for S1 and S2.

### C. S1 AND S2 IDENTIFICATION

**FIGURE 1.**
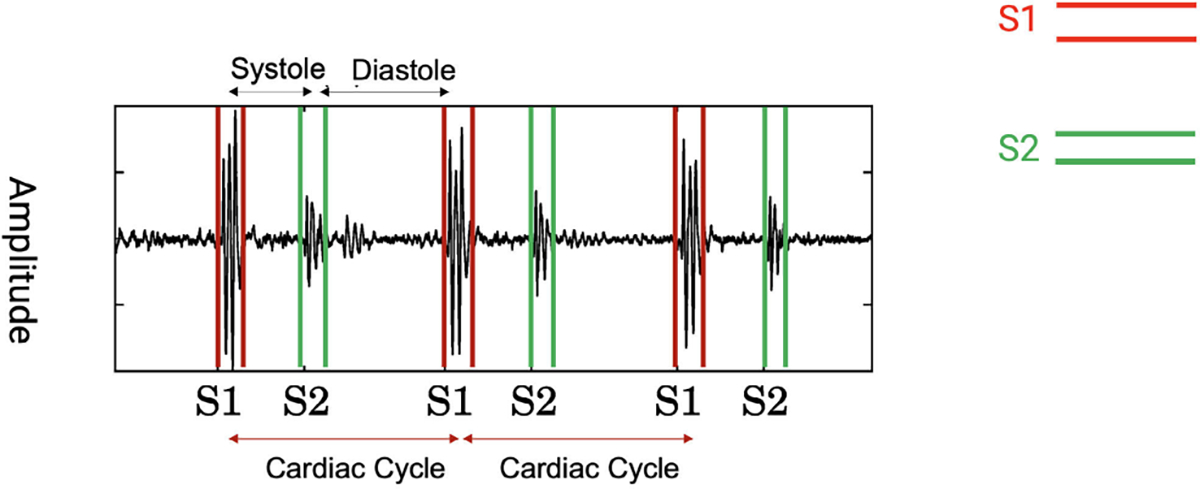
Phonocardiogram showing the recording for normal heart sounds.

**FIGURE 2.**
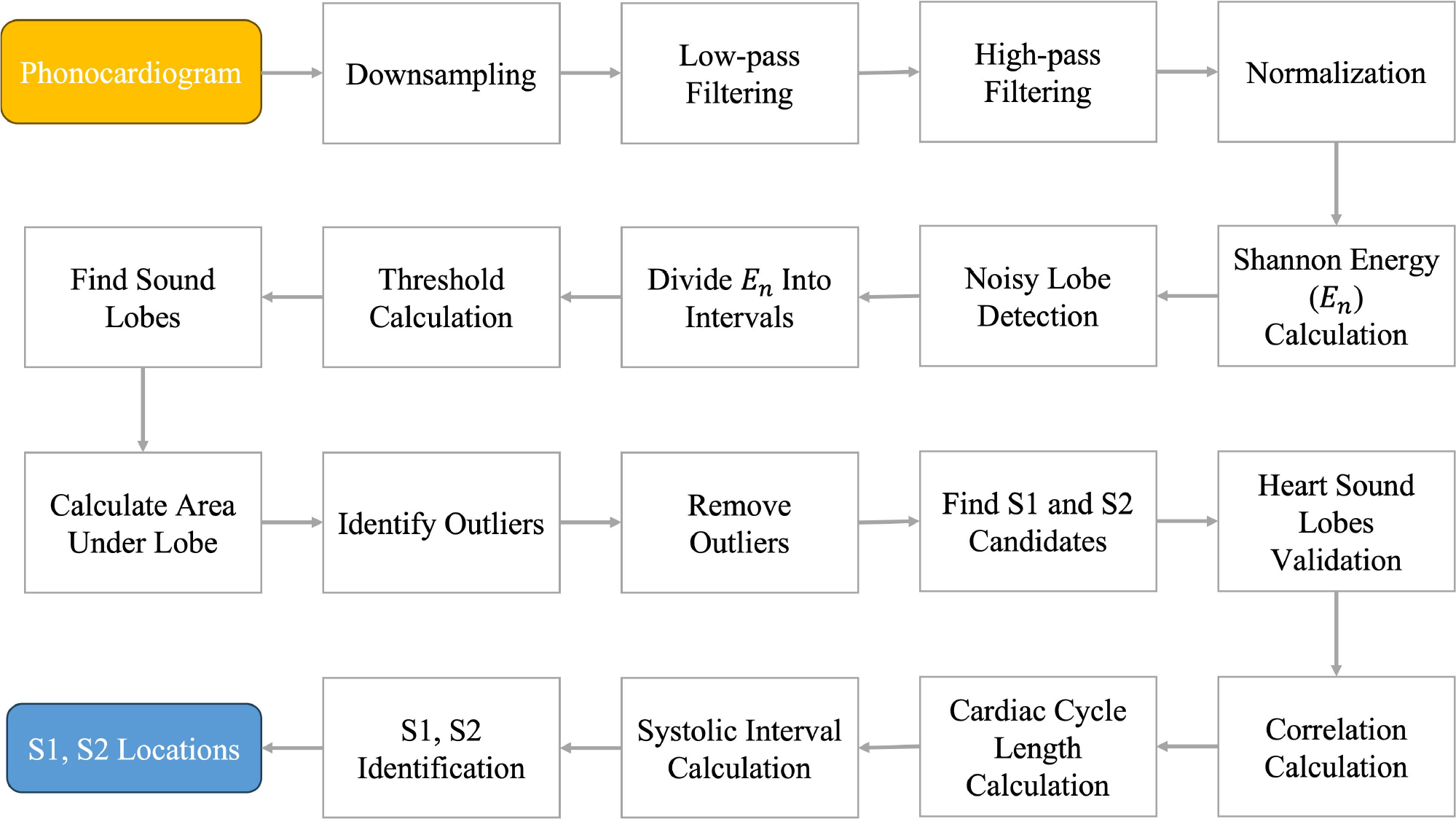
Noise-Robust HSS blockdiagram.

After validating candidate S1 and S2 sound lobes, S1 and S2 are identified sequentially using three measurements:

1. the correlation between the envelope signal $\left(E_{s}\right)$ of the possible systolic interval and the $\left(E_{s}\right)$ of the previously identified systolic interval,
2. the calculated cardiac cycle length, and
3. the calculated systolic interval.

the correlation is computed on the envelope instead of the original sound signal. The correlation on the sound signal is less reliable because the heart sounds of two adjacent cycles can have slightly different frequencies. Moreover, the variation in the beat-to-beat interval in pediatric patients is generally higher than that in adult patients. The locally estimated cardiac cycle assists the algorithm in identifying S1 and S2. The systolic interval is relatively constant compared to the diastolic interval [4], and using this knowledge, we robustly identify S1 and S2 (see Fig. 1). After identifying the first pair of S1 and S2, we search for other S1-S2 pairs in both forward and backward directions. The starting point is the sound pair with the longest interval. Generally, the diastolic interval (S2-S1) is greater than the systolic interval (S1-S2). From the first S1-S2 pair, the algorithm assesses all the possible combinations among the candidates for S1 and S2 by comparing the correlation, on the cardiac cycle and the systolic interval, between the identified S1-S2 pair and possible S1-S2 pairs. The S1-S2 pair with the best match to the previously identified S1-S2 pair is considered the true S1 and S2. The process is repeated to detect S1-S2 pairs until the start and the end of the signal are reached.

### D. NOISE-ROBUST SEGMENTATION ALGORITHM

The described Shannon energy-based algorithm works best with curated, relatively noise-free phonocardiograms. In real-world situations, however, phonocardiograms are often contaminated with bands of noise either from a child crying or rustling noise associated with the movement of stethoscope on the chest. Furthermore, this band of noise could be located anywhere within the recording. To improve the algorithm’s performance on noisy phonocardiograms, the original algorithm was enhanced to detect and crop noisy segments and perform segmentation subsequently on the noise-free recordings. First, outlier lobes are eliminated, and the noise-robust algorithm finds the noise-free segments and feeds them into the Shannon energy-based segmentation algorithm. Subsequently, the ASE is generated from the clean recordings. Each clean part of the signal is fed to the segmentation routine and then the algorithm combines the new segments with the previously found segments. Assuming the Shannon energy envelope is used to determine different lobes (i.e., contiguous segments of energy). A lobe is represented by $L_{i}$ with a start $s_{i}$ and an end $e_{i}$:

(3) $$L_{i}=\left\{E_{s}\left[s_{i}\right],E_{S}\left[s_{i}+1\right],\ldots E_{S}\left[e_{i}\right]\right\}$$

The area under each detected lobe is computed by integrating the energy envelope within the lobe boundaries. The area under a lobe can be approximated using a simple sum if the envelope is discrete:

(4) $$A\left(L_{i}\right)=\sum_{n=s_{i}}^{e_{i}}E_{S}\left[n\right]$$

The lobe areas are analyzed to identify outlier lobes. This is done by comparing each lobe’s area with the average area and standard deviation of all lobes. If there are M detected lobes, the average area $\overset{‾}{A}$ is:

(5) $$\bar{A}=\frac{1}{M}\sum_{i=1}^{M}A\left(L_{i}\right)$$

A lobe $L_{i}$ is considered noisy if its area deviates significantly from the average lobe area.

A higher z-score (deviation from the average) indicates a potentially noisy lobe. Lobes with area z-scores exceeding a predefined cutoff value (*area*_*cutoff*) are considered as noisy lobes. The standard deviation of the lobe area is computed as,

(6) $$\sigma_{A}=\sqrt{\frac{1}{M}\sum_{i=1}^{M}{\left(A_{i}-\bar{A}\right)}^{2}}$$

where:

- $\overset{‾}{A}=$ Average area of lobes
- $A_{i}=$ Area of the $i^{\text{th}}$ lobe
- M= Total number of lobes
- $\sigma_{A}=$ Standard deviation of the lobe areas

A cutoff value was set to

cutoff=2.75

The z-score is computed as,

(7) $$z_{i}=\frac{A_{i}-\overset{‾}{A}}{\sigma_{A}}$$

Then, we check if the lobe is an outlier: If $z_{i}>$ cutoff, then the lobe is considered an outlier.

The noisy lobes are recorded for further analysis. By considering the variation in lobe areas, the algorithm aims to accurately detect and isolate noisy lobes in the energy envelope. These noisy lobes can potentially correspond to artifacts, background noise, or other undesired components in the signal. Once the algorithm removed the outlier lobes, the clean signal is fed to the original Shannon energy-based segmentation algorithm. The pseudocode of the algorithm is summarized in Algorithm 1.

## IV. DATA DESCRIPTION

The performance of the noise-robust segmentation algorithm was validated on two datasets, described next.

### A. CIRCOR DATASET

The first dataset was a subset of the CirCor DigiScope Phonocardiogram dataset, released as the training dataset for the 2022 PhysioNet challenge. We refer to this subset here as the CirCor dataset. The CirCor dataset comprised 797 heart sound recordings made using Littmann 3200 electronic stethoscopes (3M Company, St Paul, Minnesota, USA) as part of two mass screening campaigns conducted in Northeast Brazil (cc2014 and cc2015). Each digital recording was 3 s-25 s in duration and was obtained from one of the four common chest locations. These recordings were distributed as recordings with murmur absent (602), with murmur present (164), or unsure (31). The gender distribution was 408 females and 389 males; and the age distribution was 591 children, 83 infants, 39 pregnant women, 78 adolescents, and 6 young adults. The ground truth S1 and S2 segmentation for these 797 recordings was initially generated by three algorithms: HSMM [65], deep convolutional neural networks [59], and adaptive Sojourn Time HSMM [64]. The results of the algorithms were examined by two expert cardiac physiologists who provided manual annotations wherever the algorithms disagreed. These files retained only the manual annotations for segmentation for the sections of recordings indicated as high-quality representative sections. The left-out sections might include both high- and low-quality sections, as suggested by Oliviera et al. [21], but were not considered because they lacked the ground truth.


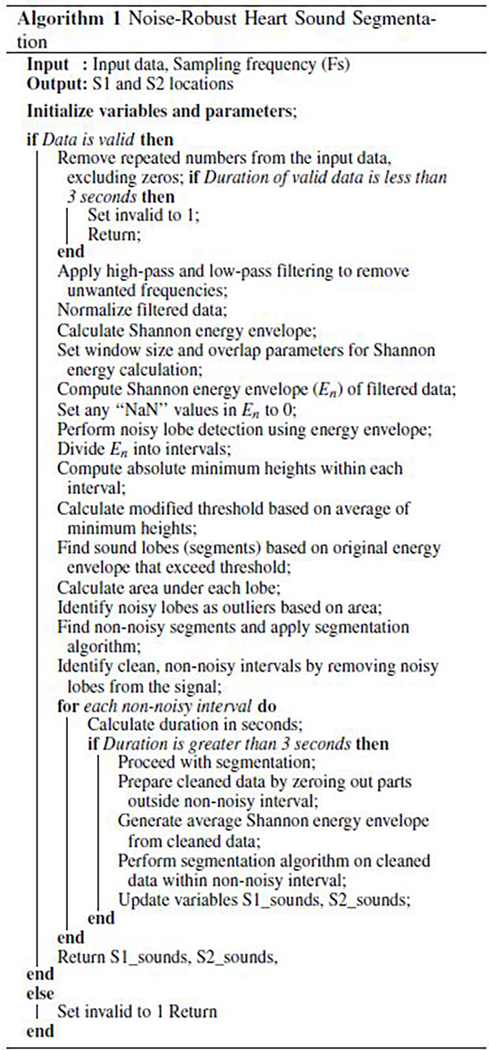


### B. CNH DATASET

The CNH dataset was compiled at Children’s National Hospital by one of the authors (R.W.D), a cardiologist with 50 years of experience. The recordings were made using a Littmann 4100 electronic stethoscope, and the dataset consisted of 1174 heart sound recordings of which 211 were innocent Still’s murmurs, 235 were normal heart sound recordings (i.e., had no murmurs), and 728 were pathological murmurs. A more detailed breakdown of the recordings by murmur type is provided in Table 4. These recordings were obtained from the same common chest locations as those for the CirCor dataset. The ground-truth murmur type for Still’s murmur recordings was provided by the same cardiologist author, and that for pathological murmur recordings was further confirmed with echocardiography. To build the S1/S2 ground truth for segmentation of CNH recordings, a MATLAB graphical user interface (GUI) was developed to allow a user to interactively annotate S1 and S2 sounds. This GUI enabled navigation between different heart sound recordings, listening to the “WAV” sound files, and displaying the corresponding waveform and the spectrogram. The GUI also enabled the cardiologist annotator to select the location of noise in the recording or annotate it as clean otherwise. Note that the segmentation algorithm was developed using only a subset of the CNH dataset comprising 257 recordings (87 Still’s murmurs and 170 non-Still’s murmurs).

### C. EVALUATION METRICS

The performance of the noise-robust segmentation algorithm was evaluated based on accuracy, sensitivity, and execution time. The accuracy was determined by calculating the average distance between the midpoints of ground truth S1 (or S2) and the midpoints of the corresponding segmented S1 (or S2) in a recording. Next, we averaged the midpoint mismatches over all S1s and S2s across all recordings. This metric provided the mean separation between the ground truth and the segmented heart sounds.

Sensitivity was assessed by verifying if the midpoint of a segmented S1 was bracketed by the start and the end points of the ground-truth S1. If the midpoint fell between the ground-truth S1 start and S1 end, then the case was considered a true positive(TP). If the midpoint was outside the said interval, the case was noted as a false negative (FN). The same process was repeated for S2 peaks. The false positive (FP) and true negative (TN) cases could not be established here because the CirCor dataset provided ground truth only for high-quality representative parts of the heart sound recordings. Only sensitivity, as defined below, could therefore be computed for CirCor dataset segmentation.


(8) $$\mathrm{Sensitivity}=\frac{TP}{TP+FN}$$

The execution time of the noise-robust segmentation algorithm is also reported to draw comparisons on the computational complexity of our algorithm. Logistic regression HSMM, one of the algorithms used to generate the ground truth in the CirCor dataset, is considered for this comparison. All experiments were performed on a MacBook Pro with Apple M1 processor and 8G as memory, and we used the built-in MATLAB functions, TIC and TOC, to estimate the execution time. This metric is important because the final objective could be to build the automated segmentation on electronic stethoscopes or mobile devices typically, which have limited computation resources. Studying the execution time of these algorithms providevaluable insights into their performance, resource requirements, and applicability to real-world clinical applications.

## V. RESULTS

The first subsection presents the performance evaluation of the noise-robust segmentation algorithm on the CirCor dataset. The second subsection presents the performance of the noise-robust algorithm on the CNH dataset.

### A. RESULTS ON CIRCOR DATASET

On the CirCor dataset, we obtained a segmentation accuracy of 0.28 ms (+/−0.02) for S1. Considering that the typical duration of S1 (or S2) is 250 ms, the obtained accuracy represented a 0.11% error. Some studies have reported shorter durations for S1 and S2 [71], [72]. For instance, Varghees et al. reported 70–150 ms (S1) and 60–120 ms (S2) [71] and Walsh and King [72] reported 50–150 ms (S1) and 30–100 ms (S2). Using 250 ms in our algorithm allowed more candidates to be identified. If the shortest S1 duration was considered, then the error would be less than 0.56%. This demonstrated that the noise-robust algorithm had a high accuracy regardless. The same applied to S2 segmentation as the noise-robust algorithm provided an accuracy of 0.29 ms (+/−0.02) assuming the S2 duration is 250 ms.

Having demonstrated the algorithm’s accuracy, the sensitivity of the noise-robust algorithm is presented in Table 5. The noise-robust algorithm had an overall sensitivity of 97.22% when evaluated with murmur-present recordings. The sensitivity was 97.69% for recordings with no murmur. The algorithm had a sensitivity of 95.76% when evaluated on the “Unsure” murmur category. The overall sensitivity considering all recordings was 97.44%. The noise-robust segmentation algorithm could find more cardiac cycles than for which the ground truth existed. Those cycles were disregarded even though they seemed valid.

Table 6 shows the execution times of the logistic regression HSMM and noise-robust segmentation algorithms on the CirCor dataset. The execution time on the entire batch of 797 recordings was 870.78 s for the noise-robust segmentation algorithm. The same for the logistic regression HSMM was 3826.56 s. On average, the noise-robust algorithm took approximately 1.09 s to segment a recording while logistic regression HSMM took approximately 4.80 s. The noise-robust segmentation algorithm executed four times faster than logistic regression HSMM.

### B. RESULTS ON CNH DATASET

**FIGURE 3.**
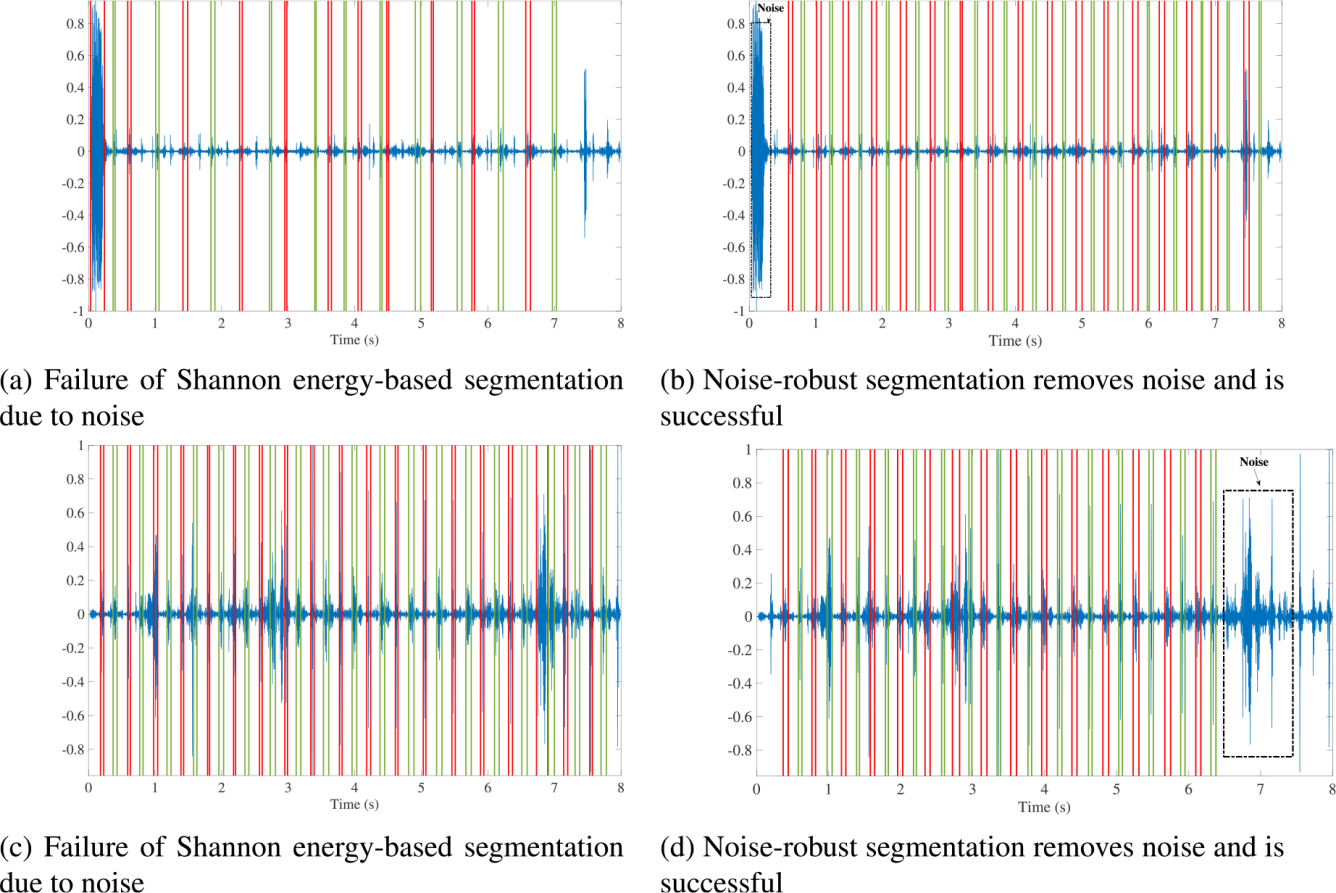
Illustration of improved segmentation using the noise-robust segmentation algorithm.

Having demonstrated high accuracy and sensitivity of the noise-robust segmentation algorithm on the CirCor dataset, our objective with the CNH dataset was to demonstrate the robustness of the algorithm against noise in a classification task. For this purpose, both the Shannon energy-based and noise-robust segmentation algorithms were testedon the CNH data. For the analysis here, the segmentation fails for a recording if the algorithm was not able to identify all the heart sound cycles available. Examples of these segmentation results are present in Figures 3 through 5.

Figure 3 (a) and (b) depict an example of a recording that was not successfully segmented because of the noise present at the start of the recording but was correctly segmented by the noise-robust algorithm and its smart cropping of the noisy segment. This recording had a loud voice (annotated in black) at the beginning of the recording. The Shannon energy-based algorithm took this noise as S1 that led to an incorrect segmentation. The noise-robust algorithm detected this noise correctly, cropped it, and then performed segmentation on a relatively clean heart sound recording. Consequently, the segmentation was correct (see Figure 3 (b)). In Figure 3 (c), we demonstrate another example of failed segmentation due to the presence of noise towards the end of the recording. In this figure, there is an overlap between S1 and S2 around 7 s into the recording. In Figure 3 (d), the noise-robust algorithm performed smart cropping of the noisy segments (from around 6.5 s to 8 s) of the recording and output only the cycles that were correctly segmented.

**FIGURE 4.**
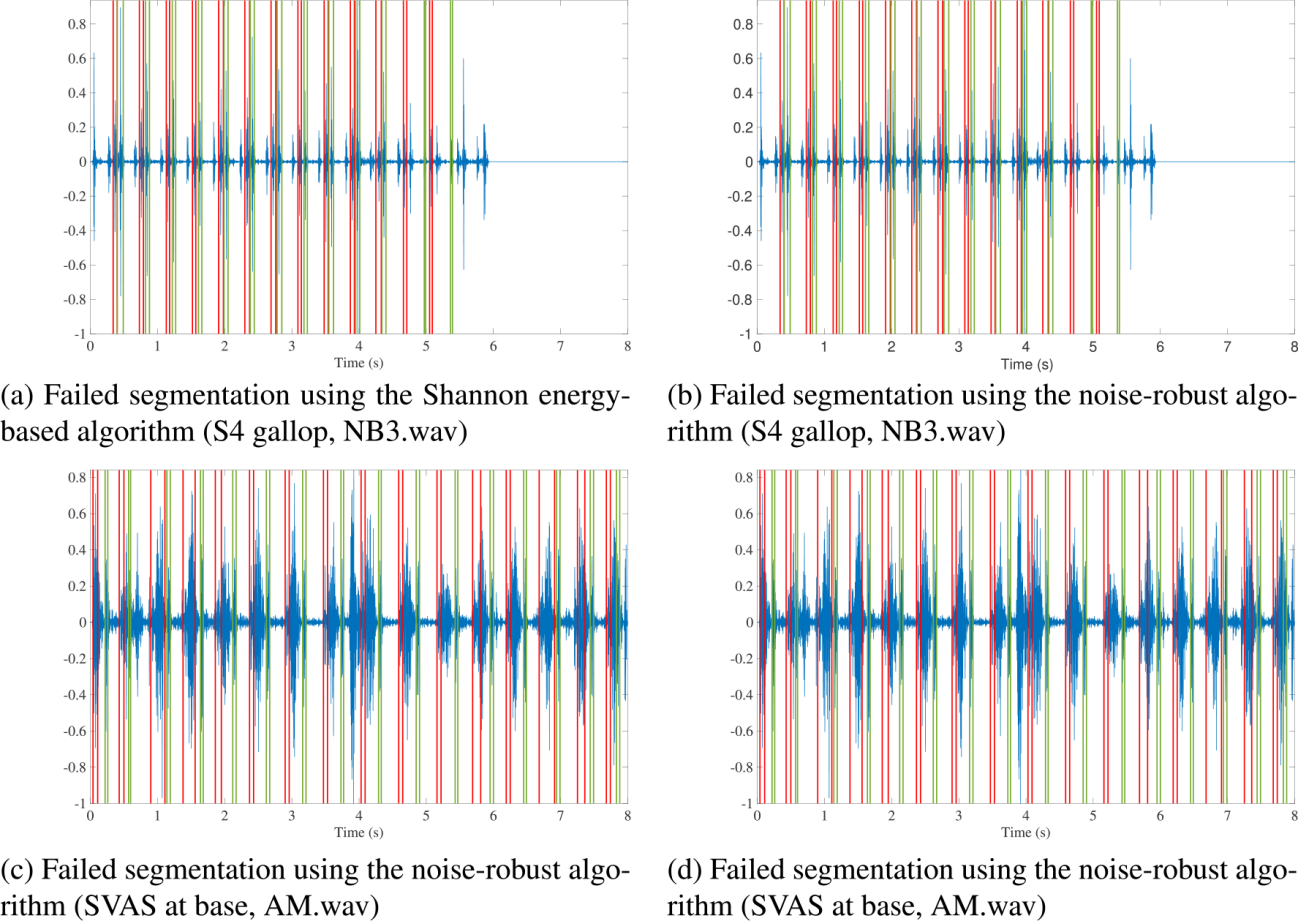
Examples of failed segmentation by both algorithms.

Figure 4 presents examples of heart sound recordings in which both segmentation algorithms did not succeed. The phonocardiograms shown here corresponded to S4 Gallop (Figures (a) and (b)). S4 Gallop is a pathological murmur in which there is an extra sound before S1. The distance of this extra sound from S1 is shorter than the distance between S1 and S2. The algorithm misidentified S4 as S1 and S1 as S2, and this led to segmentation failure for the entire recording. It is also worth mentioning that the patient had tachycardia with a heart rate of 140 beats per minute.

Figure 4 (c) and (d) correspond to a supravalvular aortic stenosis (SVAS) heart murmur recordings. This condition is a heart defect, that is a narrowing (stenosis) of the aorta, which carries blood from the heart to the rest of the body. The sound corresponding to this condition is a holosystolic murmur. Holosystolic murmurs pose a challenge not only to our algorithm but also to most other algorithms cited above except fractal decomposition methods.

**FIGURE 5.**
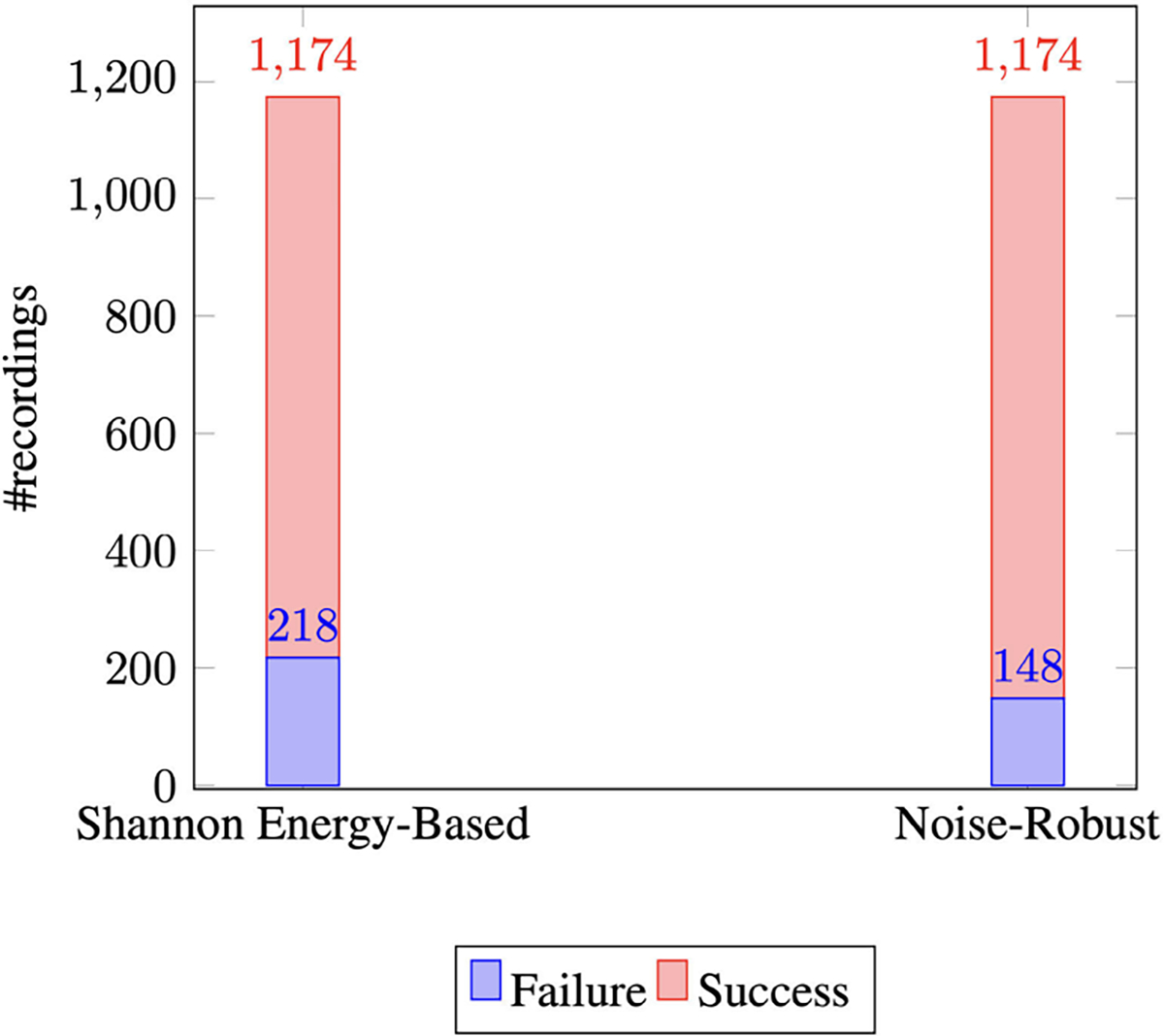
Number of segmentation failure of Shannon energy-based algorithm versus that of the noise-robust segmentation algorithm.

In a pooled analysis on the CNH dataset, Figure 5 shows that the Shannon energy-based algorithm successfully segmented 956 heart sound recordings whereas it failed in 218 of them. The noise-robust algorithm, in comparison, successfully segmented 1026 heart sound recordings and failed in a fewer number of cases (148). The noise-robust segmentation algorithm is an improvement over the Shannon energy-based algorithm segmentation by 6% in that the success rate went from 81.4% (956/1174) to 87.4% (1026/1174).

### C. DISCUSSION

The subjectivity of auscultation and the practitioners’ diminishing auscultatory skills are driving the development of computerized auscultation. Heart sound segmentation is a critical first step for identifying primary heart sounds and systolic and diastolic intervals that are important for automated heart sound analysis. Recently, heart sound segmentation has played a key role in developing artificial intelligence-assisted auscultation.

Liu et al. [19] proposed classification of ASD, VSD, PDA and combined CHD based on deep learning after heart sound segmentation demonstrating high sensitivity and specificity values. Latif et al. [73] compared several recurrent neural networks (RNNs) models’ performances for classifying heart murmurs. They divided phonocardiograms into segments of 2, 5, and 8 complete cardiac cycles. Then, for each segment, they extracted MFCCs and fed them to different RNN classifiers. Other researchers skipped completely the segmentation stage and they fed the entire signal to deep learning classifiers. Nilanon et al. [33] split phonocardiograms to sequential 5-s sections with a 1-s stride. The spectrograms and MFCCs for these sections were the input to a CNN classifier. RNNs also have been recently embedded into end-to-end approaches without employing segmentation first. Thomae and Dominik [74] proposed an end-to-end deep neural network combining 1-dimensional convolutional layers and gated recurrent unit (GRU) layers, where the phonocardiograms were entirely fed into the network. Although a few recent CNN-based techniques skip this segmentation step, there are strong reasons to believe that the performance of these models can be improved if a robust segmentation algorithm is incorporated.

Despite the existence of some studies that reported accurate heart sound segmentation, there is room for improving the performance of these methods. The performance of contemporary heart sound segmentation is still not robust and reliable. In some studies, accurate heart segmentation was achieved using electrocardiography. Expert segmentation is often considered, or combination of several segmentation algorithms has been performed to provide the ground truth for the Physionet 2022 Challenge dataset. Even when using multiple algorithms, domain experts were needed to verify and edit the output of the three algorithms. Expert annotation is tedious and time consuming. In addition, reported techniques have been mostly for adults. Segmentation of pediatric heart sounds is more challenging because of the faster heart rate and high variability. We aimed at developing effective and robust pediatric heart sound segmentation that can replace the expert heart segmentation. Previously, an algorithm based on Shannon energy was reported and tested on a curated dataset where the regions of noise were manually cropped from the phoncardiograms. The performance of this algorithm was superior to many other approaches, but its performance suffered when run on recordings corrupted with bursts of noise. The algorithm misidentified noise peaks as either S1 or S2. This motivated the development of the described noise-robust algorithm.

The noise-robust segmentation algorithm could decrease segmentation failure by 32.11% compared with the Shannon energy-based algorithm. The algorithm only failed to segment two Still’s murmur recordings, and the failure in some pathological murmurs such as holosystolic murmurs is not unexpectedasS1andS2maynotbedistinct(having murmurs that obfuscate the positions of the fundamental heart sounds). This shows the superiority of the noise-robust algorithm in dealing with noise contaminating the heart sound recordings. It is thus more practical for clinical use scenarios and can support AI research in heart murmur classification.

The noise-robust segmentation algorithm can also achieve performance comparable to the segmentation performed by clinical experts. The algorithm has been extensively tested on an independent dataset collected in a different geographic area, and the dataset includes heart sounds of infants, children, young adults, adults, and pregnant women. In some cases, our algorithm can provide heart sound segmentation which could be difficult for clinicians to perform. The noise-robust segmentation algorithm has been used for the task of Still’s murmur identification and has allowed our 5-layer convolutional neural network model to achieve 90% sensitivity and 98% specificity [11].

For comparison purposes, the results of some of the recent heart sound segmentation approaches are reported next. Renna et al. [59] proposed a CNN-based heart sound segmentation using the 2016 PhysioNet dataset [80]. The authors reported an average sensitivity of 93.9% in detecting S1 and S2 sounds. On the same dataset, Gaona and Arini [75] used Long Short-Term Memory architecture and reported an average sensitivity of 89.5%. Liu et al. [76] proposed a heart sound segmentation method that combines the time-domain analysis, frequency-domain analysis and time-frequency-domain analysis. They tested their algorithm on an authoritative heart sound database, and they showed that the boundary localization has a sensitivity of 100% and accuracy (Acc) of 99.93%. Pedrosa et al. [81] developed a segmentation algorithm based on the autocorrelation function for pediatrics, and reported a sensitivity of 89.2%. Gharehbaghi et al. [11] reported an algorithm that employed both the electrocardiogram and phonocardiogram signals for an efficient segmentation under pathological circumstances. They reported an accuracy of 97% for S1 and 94% for S2 identification. Muqing et al. [77] proposed a convolutional neural recurrent network-based on improved MFCC features and reported an accuracy, sensitivity, and specificity of 98.3%, 98.7%, 98.0%, respectively. Baghel et al. [78] proposed a CNN method for 1D time-series signals and achieved an accuracy of 98.6%. Alafif et al. [79] proposed heart rate recognition using CNN and utilized transfer learning for efficient training but achieved an accuracy of 89.5% only. Xu et al. [82] proposed a HSS algorithm based on K-Mean clustering and Wavelet transform. They reported a recognition rate of 98.02% for S1 and of 96.76% for S2. The performance of the proposed algorithm achieved a sensitivity of 97.4%, which is close to these cited methods.

Besides the performance of the algorithms, the proposed approach present several advantages. Like with the application of DL in other medical fields, the interpretability of DL in heart sound segmentation is limited. As DL models are designed to handle the complexities and nuances of large datasets, they are often too complex to comprehend or explain their failures. Therefore, it is difficult to determine why a given model may be producing a certain result or why it may be missing specific nuances in the data. The relative lack of interpretability inherent in deep learning approaches positions the proposed HSS algorithm as a more viable candidate for clinical adoption compared to deep learning-based heart sound segmentation techniques. Furthermore, the path to obtaining the regulatory clearances/approvals of traditional algorithms compared to deep learning-based ones could be less challenging as regulatory bodies, such as FDA sets a high bar to clear AI algorithms because of the lack of interpretability. The interpretability of medical algorithms plays a significant role in the process of obtaining FDA clearance. The FDA evaluates these technologies not just for their efficacy and safety, but also for the clarity and transparency of their decision-making processes. Algorithms that are more interpretable allow clinicians and regulators to understand how and why specific decisions or diagnoses are made. This transparency is crucial for trust, particularly in high-stakes medical decisions. Sensors and stethoscopes have different frequency responses as we demonstrated in [83] which could affect the performance of DL approaches and fine-tuning may be required. However, our approach is universal as it has been tested on datasets acquired with different stethoscopes such as Littmann 4100, StethAid, and others. Last but not least, medical data limitation has been a challenge to AI development and this could limit the adoption of deep learning-based approaches as they are data hungry approaches.

#### 1) SMARTPHONE APPLICATIONS

The algorithm has been effectively deployed within the StethAid platform as an iOS mobile application, leveraging the enhanced computational capabilities of modern smartphones. This algorithm has been deployed on StethAid mobile digital auscultation platform [84]. These advancements in smartphone technology facilitate the deployment of AI-based diagnostic models, particularly for heart sound analysis. Utilizing smartphone-based models for diagnostics introduces a convenient and accessible approach for patient self-monitoring. This setup significantly contributes to the early detection of cardiac anomalies, thereby streamlining timely medical intervention. Moreover, such mobile applications democratize healthcare access, transcending geographic and socioeconomic barriers. They empower individuals to conduct regular disease screenings, leveraging the ubiquity and accessibility of smartphones.

#### 2) LIMITATION OF STUDY

Directly comparing the performance of the proposed algorithms to heart sound segmentation methods is challenging due to several factors. Firstly, the diversity of test datasets used for evaluation makes a direct comparison difficult. Additionally, to fairly assess the performance of our approach against some deep learning methods, it would be necessary to train and test these architectures on the CirCor datasets and CNH dataset, which may require adjusting the hyperparameters of these architectures from their original settings. Utilizing the specified parameters without adaptation could lead to sub-optimal performance and potentially unfair comparisons.

#### 3) FUTURE RESEARCH DIRECTION

Deep learning, especially convolutional neural network-based approaches, has been explored in automated heart sound segmentation but its full potential has not been fully explored. Recently, a new type of DL has emerged based on on the concept of attention. Transformers such as [85] have achieved excellent performance on audio classification compared with CNN, but they have not been thoroughly explored in heart sound segmentation. An interesting research direction could be the exploration of the performance of transformers in automated heart sound segmentation. An attempt in a similar task has been conducted by Cheng and Sun [86] as they introduced an approach using a combination of a one-dimensional convolution (1D-Conv) module and a transformer encoder for heart sound classification and showcased remarkable accuracy of 96.4%, 99.7%, and 95.7% across three distinct datasets.

## VI. CONCLUSION

This paper presented a noise-robust segmentation algorithm based on Shannon energy envelogram that can segment primary heart sounds with high accuracy and high sensitivity. The algorithm also executes with high computational efficiency. The algorithm builds on our previous algorithm, which achieved acceptable performance but struggled in the presence of noise. The algorithm has been validated for automated segmentation of S1 and S2 in heart sound recordings of pediatric patients using two datasets: a subset of the 2022 PhysioNet Challenge dataset and locally developed dataset. This independent dataset ensured the effectiveness of the segmentation algorithm. The results indicated accurate identification of S1 and S2 when the heart sound is normal, with a murmur, with Still’s murmurs, and with a moderate pathological murmur. The incorporation of this algorithm in our murmur analysis led to fully automated identification of Still’s murmur with improved sensitivity and specificity demonstrating that an improved heart sound segmentation contributes to an improved deep learning based-heart murmur classification performance.

## Figures and Tables

**TABLE 1.** Studies on classification of cardiovascular diseases based on machine learning and deep learning, with and without heart sound segmentation.

2016 PhysioNet Challenge Top-Scoring Papers				

Reference	Segmentation	Sensitivity(%)	Specificity (%)	Classifier

Potes et al. [22]	✓	94.24	77.81	Adaboost and CNN
Zabihi et al. [23]	***X***	86.91	84.90	Ensemble NNs
Kay et al. [24]	✓	87.43	82.97	NN
Bobilo et al. [25]	✓	86.39	82.69	LR, SVM, KNN
Homsi et al [26], [27]	✓	88.84	80.48	Ensemble
Plesinger et al. [28]	✓	76.96	91.25	Prob Ass
Robin et al. [29]	✓	72.78	95.21	CNN
Abdollahpur et al. [30]	✓	76.96	88.31	NNs
Tang et al. [31]	***X***	82.20	81.49	Back propagation NN
Tschannen et al. [32]	✓	84.82	77.62	SVM
Nilanon et al. [33]	✓	76.96	85.27	LR, SVM, RF, CNN
Whitaker et al. [34]	✓	84.29	77.16	SVM
Yang et al [35]	***X***	77.49	82.87	RNN
Yazdani et al. [36]	✓	74.87	85.08	Ensemble of Classifiers
Banerjee et al. [37]	✓	80.10	79.01	RF
Singh-Miller et al. [38]	***X***	73.82	84.99	RF
Ryu et al. [39]	✓	66.63	87.75	CNN
Yang et al. [40]	✓	66.49	90.88	SVM
Bouril et al. [41]	✓	73.30	83.98	SVM
Ortiz et al. [42]	✓	78.53	78.55	LR

Post 2016 PhysioNet Challenge Papers				

Reference	Segmentation	Sensitivity(%)	Specificity (%)	Classifier

Kang et al. [15]	✓	84 – 93	91 – 99	ANN and SVM
Pyles et al. [16]	✓	78.5	92.6	Ensemble ANN
Thomson et al. [17]	✓	93	81	AI
Chobra et al. [10]	✓	76.3– 90	91.4	DL
Lv et al. [18]	***X***	97 – 89		CNN
Liu et al. [19]	✓	96 – 95	82 –80	DL
Shekhar et al. [11]	✓	90	99	CNN

**TABLE 2.** 2022 PhysioNet Challenge studies on murmur detection based on machine learning and deep learning, with and without heart sound segmentation. The entries of the tables are ranked based on the clinical outcomes defined by the challenge.

Rank	Team	*F-measure*	Accuracy	Segmentation
1	HearHeart [43]	0.619	0.801	Segmentation to patches with 7.5 overlapp
2	CUED Acoustics [44]	0.623	0.763	Yes
2	HearTech+ [45]	0.647	0.822	3s segments
4	PathToMyHeart [46]	0.686	0.778	Each patient’s recording is segmented into overlapping log mel spectrograms
5	CAU UMN [47]	0.521	0.786	No
6	Care4MyHeart [48]	0.695	0.851	Each recording is split into 5000 Segments but not cycles.
6	SmartBeatIT [49]	0.633	0.809	4-second segments
8	CeZIS [50]	0.586	0.761	No
9	Murmur Mia! [26]	0.592	0.771	Yes
11	PhysioDreamfly [51]	0.652	0.799	Unsegmented
12	Heart2Beat [52]	0.579	0.738	No [weekly labeled data+ multistage ML]
13	Listen2YourHeart [53]	0.597	0.706	No
14	Revenger [54]	0.559	0.843	No
15	matLisboa [55]	0.610	0.807	No (model produced segmentation to reduce overfitting)

**TABLE 3.** Summary of the advantages and limitations of different classes of segmentation techniques.

Category	Advantages	Limitations

Category 1: Wavelet transform [56]	• Split a signal into frequency bands • Adequate performance • Balance between time and frequency	• Works well only with non-stationary signals • Are not always guaranteed to be robust when applied to heart sounds collected in different conditions and with different devices
Category 2: Fractal decomposition [57]	• Work in time domain • Able to separate murmurs that act very similar to fractals	• Tested on a limited dataset of 23 heart sound recordings.
Category 3: Hilbert transform [58]	• Split a signal into frequency bands • Works well for non-linear and non-stationary signals • Estimate understated changes in frequencies	• Do not provide sufficient performance to allow analysis of wideband signal
Category 4: HSMM [64], [65]	• Sojourn time enables effective heart sound segmentation • Robustness to training testing dataset variability	• The solution involving the use of an HMM and the use of the Viterbi algorithm requires the observation of the entire sequence before starting the decoding procedure • It has a high computational complexity which is of the order O(4N), where N is the number of samples of the considered PCG signal
Category 5: Deep learning [12], [59]	• Potential for improved performance and continual improvement with more training data • Robustness to noise and artifacts • Automatic feature learning • End-to-end approach • Adaptability to various heart sound patterns	• Possible hardware/device bias • Insufficient training data • S1/S2 annotation challenges • Generalizability across different populations and conditions • Lack of interpretability may raise concerns in clinical applications • Computational requirements can hinder the deployment and real-time application of deep learning-based segmentation algorithms in resource-constrained environments such as mobile or embedded systems.
Category 6: Hybrid methods [60], [66]	• Accurate segmentation • Benefits from QRS for nearly perfect segmentation	• Requires additional hardware and software • Some pathological conditions may cause alterations of the PCG waveforms that could compromise the reliability of this method • External electrocardiography is not as widely available as electronic stethoscopes
Category 7: Shannon energy [61], [62]	• Superior segmentation accuracy • Computational efficiency • Diminish unwanted signal peaks • Work in time domain and does not require split in frequency bands • Interpretable results • Potential for real-time implementation • Low data requirements • Applicability to a wide range of heart sound recordings (PCG and ECG)	• May be sensitive to noise present in heart sound recordings such as environmental noise, artifacts, or other sources of interference can affect the accuracy of the segmentation results • Performance on atypical heart sounds may pose challenge

**TABLE 4.** The distribution of recordings by murmur type in the CNH dataset.

Class	Count
Still’s Murmur	211
No Murmur (Normal)	235
VSD-Pathological Murmur	199
PS-Pathological Murmur	78
Venom Pathological Murmur	65
PDA-Pathological Murmur	50
AS Pathological Murmur	34
TR-Pathological Murmur	32
MR-Pathological Murmur	24
RVOT Pathological Murmur	22
ASD Pathological Murmur	19
AI-Pathological Murmur	12
PR-Pathological Murmur	12
Other-Pathological Murmur	181
Total	1174

**TABLE 5.** Sensitivity of the algorithm for S1 and S2 segmentation.

Heart sound	Number of cycles	TP	FN	Sensitivity (%)
Murmur Present	19488	18946	542	97.22
Murmur Absent	68346	66766	1580	97.69
Unsure	6693	6397	296	95.76
Total	94527	92109	2418	97.44

**TABLE 6.** Comparison of execution times of segmentation algorithms on the CirCor dataset.

Algorithm	Execution time on CirCor dataset
Logistic Regression HSMM	3826.56 s (1.06 h)
Noise-robust HSS	870.78 s (0.24 h)

**TABLE 7.** Performance of deep learning-based HSS techniques compared to Noise-Robust HSS method.

Approach	Performance
Renna et al. [59]	Sensitivity=93.9%
Gaona et al. [75]	Sensitivity=89.5%
Liu et al. [76]	Sensitivity=100%
Gharehbaghi et al. [66]	Accuracy=97% for S1 and Accuracy=94% for S2.
Muqing et al. [77]	Accuracy= 98.3%, Sensitivity=98.7%, and Specificity= 98.0%
Baghel et al. [78]	Accuracy=98.60%
Alafif et al. [79]	Accuracy= 89.5%
Proposed Approach	Sensitivity=97.4%
